# Combating Malaria: Targeting the Ubiquitin-Proteasome System to Conquer Drug Resistance

**DOI:** 10.3390/tropicalmed10040094

**Published:** 2025-04-03

**Authors:** Bazgha Sanaullah, Nguyen Van Truong, Tuyet-Kha Nguyen, Eun-Taek Han

**Affiliations:** Department of Medical Environmental Biology and Tropical Medicine, School of Medicine, Kangwon National University, Chuncheon 24341, Republic of Korea

**Keywords:** malaria, drug resistance, ubiquitin-proteasome system (UPS), artemisinin, inhibitors, antimalarial drugs

## Abstract

Malaria primarily affects developing nations and is one of the most destructive and pervasive tropical parasite infections. Antimalarial drug resistance, characterized by a parasite’s ability to survive and reproduce despite recommended medication doses, poses a significant challenge. Along with resistance to antimalarial drugs, the rate of mutation a parasite undergoes, overall parasite load, drug potency, adherence to treatment, dosing accuracy, drug bioavailability, and the presence of poor-quality counterfeit drugs are some of the contributing factors that elicit opposition to treatment. The ubiquitin-proteasome system (UPS) has become a promising drug target for malaria because of its central importance in the parasite’s life cycle and its contribution to artemisinin resistance. Polymorphisms in the Kelch13 gene of *Plasmodium falciparum* are the best-known markers for artemisinin resistance and are associated with a highly active UPS. Certain proteasome inhibitors, which are the other key players of the UPS, have demonstrated activity against malarial parasites and the ability to work with artemisinin. This work describes how, through targeting the UPS, the greater effectiveness of antimalarial drugs—especially where there is strong resistance—can be achieved, which contributes to overcoming the drug resistance phenomenon in malaria.

## 1. Introduction

Malaria is a life-threatening disease caused by a parasite of the *Plasmodium* genus and is a significant global health issue [1,2,3]. Indeed, malaria claims the lives of several hundred thousand people annually, mostly young children in Sub-Saharan Africa [4,5,6,7]. However, the emergence of drug resistance against *P. falciparum* presents a major obstacle on the path of disease control [8,9]. Rising medication resistance has increased the need for new treatments, effective not just for acute and severe infections with elevated blood parasite counts but also on different parasite stages to interrupt transmission [10]. Thus, identifying drug-targeting mechanisms at various parasite stages will be crucial in eradicating malaria [11].

Recent studies have demonstrated the ubiquitin-proteasome system (UPS) as a suitable target for the discovery of new antimalarial therapeutics [12,13]. The UPS is necessary for controlling protein degradation and turnover in all eukaryotic cells, including malarial parasites [14,15]. A new perspective in the control of malaria is the targeting of important signaling pathways and mechanisms that are essential for the survival and replication of the malarial parasite [16].

Blocking the UPS pathways generates multiple points of attack on the parasite, enhances the effectiveness of antimalarial drugs, and decreases drug resistance [17]. Mapping out these pathways will help to create new cures against malaria, one of the biggest public health challenges [18]. The UPS is a highly conserved cellular pathway among all *Plasmodium* species and provides a broad-spectrum target for antimalarial development [19,20]. Moreover, the UPS participates in multiple cellular processes and pathways that are critical for *Plasmodium* survival and development, involving protein quality control, cell cycle regulation, and immune evasion [21]. All of the processes which are Medical Complex Mechanism Obstruction (MCMO) can be disrupted by acting on the UPS, ultimately killing the parasite and preventing the development of malaria [22]. Previous work has identified specific proteins and signaling pathways for targeted attack within the malarial parasite’s ubiquitome [18].

These findings opened up new pathways for designing new antimalarial drugs that target the UPS selectively [12]. These drugs provide hope not only for malaria treatment, but also as a solution for the increasing issue of drug resistance [20].

## 2. Methodology

**Figure 1:**
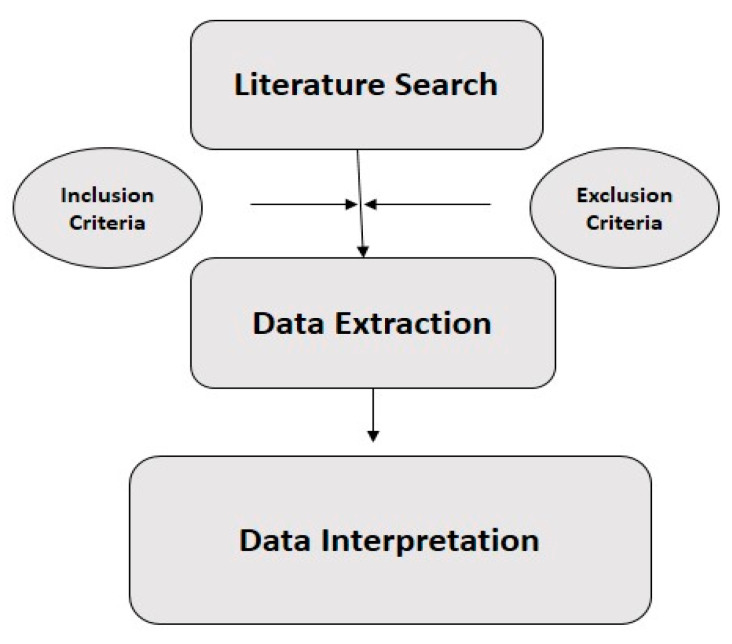
The methodology utilized in our review paper.

This review aims to provide a comprehensive overview of the ubiquitin-proteasome system (UPS) as a potential target for combating malaria, with a particular focus on drug resistance mechanisms and the development of proteasome inhibitors (Figure 1). A broad range of relevant literature was analyzed to synthesize current findings, identify research gaps, and suggest future directions.

### 2.1. Literature Search

A thorough search was performed using electronic databases such as PubMed, Scopus, Web of Science, and Google Scholar. Keywords used included “new interventions in malaria”, “ubiquitin-proteasome system”, “proteasome inhibitors”, “drug resistance in malaria”, and “UPS inhibition in *P. falciparum*”.

### 2.2. Inclusion and Exclusion Criteria

Articles published in peer-reviewed journals were included, focusing on recent advances in proteasome inhibitors for malaria treatment. Studies on drug resistance mechanisms, molecular pathways, and clinical trials were prioritized. Non-English articles and those lacking relevant data were excluded.

### 2.3. Data Extraction

Key information from selected studies was extracted, including details on drug mechanisms, in vitro and in vivo findings, clinical outcomes, and potential drug resistance challenges. Data were then synthesized to provide a comprehensive overview of the current knowledge.

### 2.4. Interpretation

Findings were critically analyzed to identify gaps in the knowledge, challenges in current therapies, and opportunities for future drug development.

## 3. Results Interpretation

### 3.1. Current Antimalarial Drug Targets

Antimalarial treatments currently include artemisinin-based combination therapies (ACTs) such as artemether–lumefantrine (AM/LM), artesunate–amodiaquine (AS/AQ), and dihydroartemisinin–piperaquine (DHA/PQ) that are quite effective against the *falciparum* malaria [23]. These drugs function by attacking the malarial parasite at various stages of its life cycle within the human body, which increases the chances of successfully eliminating the infection [24].

Artemisinin and its derivatives create free radicals that harm the proteins and membranes of the parasite [25]. Lumefantrine, amodiaquine, and piperaquine interfere with the parasite’s capacity to detoxify heme, which is a toxic by-product produced during its metabolism of hemoglobin in the host’s bloodstream [26]. By blocking this detoxification process, these drugs result in the accumulation of toxic heme in the parasite, ultimately causing its death [27]. Other drugs, like chloroquine, have been pivotal therapies for decades. However, our understanding of chloroquine resistance has improved, with evidence pointing to alterations in pH within the digestive vacuole of *P. falciparum* as being linked to resistance to the drug [28]. Mefloquine, another antimalarial medication, acts by targeting the 80S ribosome of *P. falciparum*, and disrupts the heme detoxification pathway by interfering with the ability of parasites to metabolize heme, thereby inhibiting protein synthesis. Although mefloquine is effective, it is associated with neuropsychiatric side effects, and resistance has been reported, which underscores the need for continued research on its efficacy and safety [29]. Together, these mechanisms constitute the intricate strategy by which antimalarials combat the malarial parasite, which continually evolves alongside the development of drug resistance.

### 3.2. Possible Next-Generation Therapies

**Figure 2:**
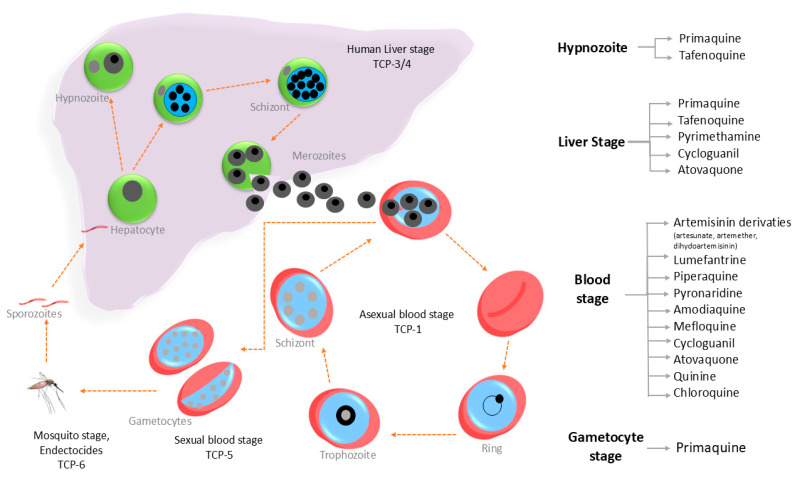
Antimalarial drugs targeting different stages of the *Plasmodium* life cycle.

The target candidate profile (TCP) specifies the essential criteria a chemical compound must meet to be considered suitable for malaria therapy [30]. Figure 2 illustrates the various antimalarial TCPs and provides an overview of the *Plasmodium* life cycle. The target product profile (TPP) defines the minimum and optimal requirements for a combination product that works well in the field and offers advantages over standard care. Target product profiles (TPPs) are categorized into two primary groups, each addressing distinct therapeutic requirements. The first category, TPP-1, prioritizes the development of novel drug combinations for the treatment of uncomplicated malaria. However, specific challenges remain, including the necessity for alternative therapies for severe malaria, where oral formulations are less effective, and for interventions targeting asymptomatic infections across broader populations, particularly dormant parasites in *P. vivax* malaria.

TPP-2 focuses on two main strategies: chemoprevention, which involves administering complete treatment doses to individuals in extremely prevalent regions to reduce transmission, and prophylaxis, where drugs are given to asymptomatic individuals at risk. Chemoprevention is crucial in the absence of a highly effective vaccine, protecting groups such as travelers, migrants, pregnant women, young children, and those with comorbidities like anemia or sickle cell disease. However, with a significant proportion of malaria cases taking place during the rainy season and the rising threat of resistance to SP-amodiaquine, new medications are desperately needed for seasonal malaria chemoprevention [31]. Identifying alternatives to SP-amodiaquine requires considering factors such as drug resistance, safety, pharmacokinetics, availability, and cost. The ideal alternatives would be two or more potent antimalarial compounds with strong safety profiles, distinct mechanisms of action, long-lasting effects, and compatible pharmacokinetic characteristics for monthly dosing in seasonal malaria chemoprevention (SMC). Additionally, the development of safe, long-acting injectables could enhance SMC, if well distributed, and offer prolonged protection [31,32].

Recent research has highlighted the urgent need for novel therapeutic targets to combat malaria due to an increase in drug resistance. The unique biology of the *Plasmodium* parasite offers promising avenues for identifying new therapeutic targets [33]. It is increasingly important to investigate these novel targets as resistance makes conventional drugs less effective [34].

### 3.3. Emergence of Drug Resistance Against Antimalarial Drugs

The World Health Organization (WHO) describes antimalarial drug resistance as the capacity of a parasite strain to persist and/or reproduce despite being exposed to drug doses that meet or exceed the recommended levels, as long as the drug is properly absorbed and reaches the site of action. This resistance occurs when genetic mutations or amplifications in the parasite result in a decreased sensitivity to the drug [35].

Antimalarial drug resistance arises due to genetic changes, including single nucleotide polymorphisms (SNPs) or gene amplification, which alter protein structure or function. This poses a significant challenge for treatment, leading to continuous research to identify effective solutions [17]. The rise in resistant parasite strains highlights the urgent need for the ongoing development of new compounds. Currently, novel drugs with various modes of action are progressing into clinical trials to solve this problem [36].

Several factors contribute in the drug resistance, such as the following: (1) Parasite mutation rate: Higher mutation rates increase the chances of genetic changes that lead to resistance. (2) Overall parasite load: A larger population of parasites creates more chances for resistant strains to develop and proliferate. (3) The strength of drug selection: How effective a drug is at killing parasites affects the selection pressure for resistant strains. (4) Treatment compliance: Inconsistent or incomplete treatment not only fails to eliminate parasites, but also allows resistant strains to survive and spread. (5) Poor adherence to malaria treatment guidelines: Not following treatment protocols can result in inadequate dosing or the use of inappropriate drugs, increasing the development of resistance [37].

Improper dosing, poor pharmacokinetic properties, and the presence of counterfeit drugs can lead to insufficient drug exposure to parasites [38]. Poor-quality antimalarial medications, including fake products that do not contain active pharmaceutical ingredients (APIs), can worsen resistance by increasing the chances of hyperparasitemia, recurrence, and hypergametocytemia. Furthermore, using incorrect APIs, like replacing halofantrine with artemisinin, can also play a role in developing resistance. Without chemical analysis, such substitutions may go unnoticed by investigators but not by parasites [39,40]. Antimalarial drugs commonly target biological pathways in *Plasmodium* parasites, such as heme detoxification in the digestive vacuole, folate and pyrimidine biosynthesis, and electron transport in the mitochondrion. Research conducted on the use of aryl amino alcohols like quinine, lumefantrine, and mefloquine in Southeast Asia has demonstrated that changes in the copy number of *pfmdr1*, as well as sequence variants of *pfcrt* and *pfmdr1*, can impact the parasite’s susceptibility to these drugs [41]. Unlike other diseases such as tuberculosis and acquired immunodeficiency syndrome, malaria drug resistance mechanisms are distinctive. The parasite can develop resistance by directly altering the target cell for the drug. In malaria, resistance is primarily caused by increased, non-specific drug efflux through the activation of multidrug resistance (MDR) transporters. However, it is important to emphasize that MDR transporters are not the main method of resistance in malaria [42].

The antimalarial effect of artemisinin arises from its distinct trioxane structure, which includes an endoperoxide bond. Clinically, semi-synthetic derivatives such as artemether, artesunate, and dihydroartemisinin are often preferred because of artemisinin’s low solubility [43]. Artemisinin combination therapy (ACT) is now recommended for malaria treatment because of the emergence of drug resistance from monotherapy for patients in endemic areas. Commonly used combinations include artemether–lumefantrine (Coartem), artesunate–amodiaquine (Coarsucam), artesunate–mefloquine (Artequin™), artesunate–sulfadoxine–pyrimethamine (Fansidar), and dihydroartemisinin–piperaquine (Eurartesim). These combinations represent the current gold standard regimes for malaria treatment.

Artemisinin and its derivatives demonstrate a rapid onset of action but are quickly cleared from the body, with a half-life ranging from 0.5 to 1.4 h. Therefore, they must be paired with drugs that have slower clearance rates to ensure the effective elimination of residual parasites [44]. Artemisinin and its derivatives are often coupled with other antimalarial drugs for two reasons: first, to prolong the half-life of artemisinin and its derivatives, and second, to prevent the development of resistance [45].

#### Resistance to Artemisinin and Other Therapeutics

Since asymptomatic people are reservoirs for the spread of malaria, artemisinin can help to lower the parasite burden in them and has a long-lasting effect on chloroquine-resistant malarial parasites [17]. But in many places, resistance to artemisinin and its derivatives is developing, which makes treating malaria extremely difficult [46]. Artemisinin-based medication resistance is undoubtedly developing in Asia’s Greater Mekong Subregion [32]. To delay the spread of resistance and lessen the chance that resistance may develop to combinations including an artemisinin derivative now in use, it becomes essential to combine two or more component medications as the resistance to each new malaria medication evolves [47]. Recent reports from Equatorial Guinea, one of the African countries, have indicated that some *P. falciparum* isolates are resistant to artemisinin [48,49].

Artemisinin and its derivatives, including artesunate, artemether, and arteether, are strong, quick-acting medications that quickly reduce parasitemia in the first few days of cure. Mass spectrometry studies have demonstrated that artemisinin can alkylate heme, which results in the endoperoxide bridge breaking down and the creation of free radicals with a carbon center. Because of these radicals, artemisinin is extremely toxic to malarial parasites, which are its primary target [50].

Many proteases in the malarial parasite-infected host break down hemoglobin to liberate peptides and amino acids that are essential to the parasite’s growth. Additionally, this procedure makes room in its digestive vacuole for hematin to accumulate, which may be harmful to the parasite. Certain proteins or enzymes are the molecular targets of artemisinin. According to in vitro research, artemisinin alkylates hemoproteins such hemoglobin, cytochrome c, and catalase, but not free globin [51].

*Pf*ATP6 is another molecular target of artemisinin. The parasite Ca^2+^-carrying ATPase called sarco/endoplasmic reticulum membrane calcium ATPase (SERCA) is inhibited by artemisinin. By actively concentrating Ca^2+^ into membrane-bound storage, SERCA lowers cytosolic free calcium concentrations, which is an essential process for cellular survival [31]. Artemisinin builds up in neutral lipids within the parasite membrane, causing damage to the membrane [32]. Artemisinin’s efficacy as an antimalarial drug is due to its ability to rapidly reduce parasitemia by targeting and alkylating hemoproteins and inhibiting vital enzymes such as SERCA. Its accumulation within parasite membranes further contributes to its antimalarial activity by causing membrane damage.

A molecular indicator for artemisinin resistance in *P. falciparum* malaria, *P. falciparum* Kelch 13 (*Pf*K13, Genbank Access. No. PF3D7_1343700 or PF13_0238) was identified from nation-wide surveillance of isolates of the GM Subregion of Asia [52]. *Pf*K13 is expected to act as a cullin E3 ligase substrate adapter. In addition to being a redox sensor, its probable substrate is *P. falciparum* phosphatidylinositol 3-kinase (*Pf*PI3K) [8]. Reduced interactions between artemisinin and *Pf*PI3K are caused by the mutant K13 [53]. *Pf*K13 propeller gene alterations are linked to artemisinin resistance, according to in vitro and in vivo research conducted in endemic African nations and other parts of Southeast Asia [54]. This indicates that the greater level of acquired immunity that is common in Africa as a result of recurrent exposure to *P. falciparum* is said to have a limited influence. Drug-resistant illnesses can be controlled with the help of this exposure, which boosts host immunity [55].

Resistance to the antimalarial drug chloroquine has been well studied. This resistance is caused by changes in pH levels within the digestive vacuole of *P. falciparum*, which prevent the drug from accumulating in the part of the parasite cell where it functions [56]. Additionally, there have been accounts of resistance to pyrimethamine, another antimalarial, due to changes in a particular protein that this drug targets [57]. It is essential for malaria control strategies to monitor and prevent resistance to antimalarial drugs. The resurgence of malaria in areas where it was once under control is often due to the spread of drug-resistant strains [58].

### 3.4. Ubiquitin Proteasome System (UPS) as an Antimalarial Drug Target

The UPS shows great potential as a drug target because it is conserved across various *Plasmodium* species and plays a significant role in many cellular processes important for the malarial parasite’s survival. New drugs that target the UPS may address various resistance mechanisms and offer a broad-spectrum approach for fighting various malarial parasites [8,18]. Further investigation of the *Plasmodium* ubiquitome has provided valuable insights for drug development, highlighting potential proteins and pathways within the UPS that could be targeted to combat the parasite [59]. These findings mark significant progress in the battle against drug-resistant malaria. Essentially, examining the UPS presents a promising path for developing new antimalarial treatments. By focusing on these innovative targets, researchers hope to identify more effective therapies that address the ongoing challenges of drug resistance, ultimately improving treatment outcomes for malaria [8].

Recent research shows how blocking the UPS could lead to breakthroughs in antimalarial treatment strategies [60]. The UPS protein degradation system is both precise and crucial for every eukaryotic organism, including the *Plasmodium* genus. Targeting the components of UPS degradation systems, like the proteasome, has shown positive activity against malaria [61]. These inhibitors impair a parasite’s ability to manage necessary proteins at the molecular scale which leads to cell death. Research shows some of the more selective deubiquitinase (DUB) inhibitors can suppress intraerythrocytic malarial parasite growth in vitro and in vivo (Table 1). These DUB inhibitors also improve the effectiveness of artemisinin for treatment which is particularly promising for combination therapy [18]. Protein turnover is just one of the biological processes controlled by ubiquitin within the parasite which also includes modulating immune responses and cellular signaling. This indicates that UPS inhibitors have the potential to disrupt various aspects of the parasite’s growth and development, offering a versatile approach to combat malaria [62].

#### 3.4.1. Structure and Composition of Plasmodium UPS

**Figure 3:**
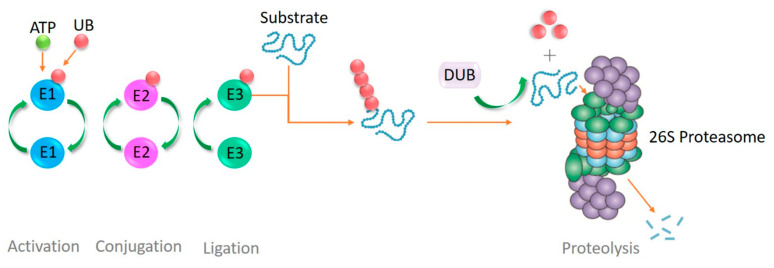
Ubiquitin proteasome system.

The *Plasmodium* UPS stands out from the other organisms studied for its distinctive combination of the conserved 26S proteasome found in eukaryotes, along with an HsIV proteasome typical of prokaryotes, and a caseinolytic protease (CIp) originating from cyanobacteria [71]. A post-translational alteration known as ubiquitylation occurs when ubiquitin (Ub) covalently binds to the target protein [18]. Three kinds of enzymes—E1 (Ub-activating enzyme), E2 (Ub-conjugating enzyme), and E3 (Ub ligase)—must work in unison to complete this process [72]. In order to facilitate the transfer of Ub from the E2 ligase to the final substrate, E3 ligases are principally responsible for determining substrate specificity [73]. Additionally, DUB activity can reverse the ubiquitylation process [74]. The amount of particular ubiquitylated proteins within the cell is influenced by the coordinated interaction between the process of ubiquitylation and deubiquitylation, which controls the activation or deactivation of specific functions (Figure 3) [75]. The proteasome, a central component of the UPS, is a sizable protein assembly comprising multiple catalytic protease subunits [76]. This large barrel-shaped protein structure is made up of a 19S regulatory particle (RP) and a 20S core particle (CP). In *Plasmodium*, all anticipated elements of the 26S proteasome have been detected and are active throughout its life cycle [77]. The CP forms a barrel-shaped complex comprising four stacked rings. Each ring consists of seven α and seven β subunits, arranged in an “αββα” configuration [78]. Within each inner ring of the CP, three β subunits (β1 (caspase-like activity), β2 (trypsin-like activity), and β5 (chymotrypsin-like activity)), each catalytically distinct, feature active sites lining the central lumen. Together, they create a proteolytic chamber where substrate degradation occurs [79].

The investigation of drugs known for their inhibition of the proteasome in myeloma for their potential application in targeting the malarial proteasome presents a promising avenue in malaria research [80]. These drugs, which were developed and optimized for their efficacy in cancer treatment, exhibit a unique ability to disrupt protein degradation pathways essential for cell survival [81]. Leveraging these drugs for malaria treatment capitalizes on their mechanism of action, which targets a fundamental biological process shared by both cancer cells and malarial parasites (Table 2). Additionally, these drugs have undergone extensive preclinical and clinical testing, providing valuable insights into their safety profile, pharmacokinetics, and potential side effects. Repurposing them for malaria therapy could expedite the drug development process, potentially leading to faster approval and deployment for clinical use [82].

#### 3.4.2. Ubiquitination of Subjected Proteins

The 76 amino acid carboxy-terminal of the ubiquitin protein is attached to surface-exposed lysine residues of redundant or misfolded proteins to start the process of protein breakdown. This tagging serves to mark the proteins for degradation by the proteasome [91]. Substrates earmarked for degradation necessitate a ubiquitin chain comprising a minimum of four residues [104]. Proteins can undergo mono-ubiquitination at one or more sites without being earmarked for degradation. Instead, they may participate in various cellular signaling processes such as DNA repair, endocytosis, or trafficking [105]. The proteolytic cleavage of Ub fusion proteins or degradation of poly-Ub proteins can yield Ub with a molecular weight of 8.5 kDa from the cell [91].

The Ub open reading frame is repeated five times in a row in *P. falciparum’s Pf*pUb gene, which codes for poly-ubiquitin [91,106]. *Pf*pUb is expressed at every stage of the life cycle, although intraerythrocytic parasites have a noticeable preponderance of it [107,108,109]. *P. falciparum* possesses two additional genes that encode Ub-fusion proteins: the uba52 homolog L40/UBI and S31/UBI [91,110]. There are also other Ub-like proteins (ULPs) in the genome, such as SUMO, Hub1, Urm1, and Atg8 homologs [110]. ULP-conjugated proteins primarily serve regulatory functions, distinct from proteasomal degradation pathways. Ubiquitination proceeds via a three-enzyme cascade: E1 (Ub-activating), E2 (Ub-conjugating), and E3 (Ub-ligase). E1 forms a thioester bond with Ub, transferring it to E2. E3 then facilitates Ub transfer from E2 to substrate lysine residues. While E1 and E2 are non-specific, E3 enzymes exhibit high substrate specificity, determining ubiquitination targets [73]. E3 ligase inhibitors showed potent antimalarial activity against *P. falciparum* strains (*Pf*D6 and *Pf*W2) with low cytotoxicity to mammalian Vero cells, blocking parasite development at the **trophozoite and schizont stages** [111]. In the presence of additional cofactors or by a unique substrate recognition domain, they provide substrate selectivity. Thus, E3 ligases can be divided into four major groups: the U-box E3 enzymes, RING, PHD, and HECT [112]. Researchers have found 54 putative E3 ligases, 14 putative E2-like enzymes, and 8 putative E1-like enzymes in *P. falciparum* (Table 3) [20,110]. Few details are known at this time about how these enzymes act in malarial parasites. There is, however, a homolog for the 13 Ub-conjugating enzymes in *P. falciparum* (*Pf*UBC13). The protein kinase *Pf*PK9 has been shown to regulate *Pf*UBC13 activity [113]. Recently, ubiquitination activity and an essential function for three parasite-specific E3 ligases were reported by Le Roch and associates [20]. Given their notable difference and essential function in the malarial parasite, the authors hypothesized that these enzymes might be a distinct class of therapeutic target. Recent research on the human E3 ligase MDM2 and the small chemical inhibitor MI-63 have provided evidence in favor of this idea by showing that targeted inhibition of E3 enzymes is feasible [114]. The movement of ubiquitinated substrates to the proteasome is facilitated by shuttle proteins. Rpn10 appears to function as a substrate shuttle in the cell and is also present there without being associated with any proteasomes. For this function, other Ub-binding proteins that are not part of the proteasome, like Rad23, Dsk2, or Ddi1, also act as shuttles [115,116,117]. These proteins have both an Ub-binding domain (UbA) and an Ub-like domain (UbL) that can bind to the proteasome’s Ub receptors [118]. With sequence identities of 29%, 27%, and 34%, orthologs of Rad23, Dsk2, and Ddi1 have been found in *P. falciparum* as PF10_0114, PF11_0142, and PF14_0090, respectively. These matches have matching *p*-values of 1.0 × 10⁻^24^, 5.2 × 10⁻^34^, and 3.5 × 10⁻^32^. Since transporting Ub-tagged proteins to the proteasome for proteolysis is crucial, drugging the shuttle proteins may be a tempting way to interfere with the UPS. On the other hand, nothing is known about their appropriateness as pharmacological targets. Girolline is one substance that has been linked to preventing Ub-tagged proteins from being delivered to the proteasome [119]. But the exact mechanism by which girolline works is not fully understood, and it may also include translation termination in malarial parasites [120,121].

#### 3.4.3. Protein Deubiquitination

Ubs are separated from the substrate to facilitate further ubiquitination cycles prior to proteasome destruction. DUBs, which are proteases in charge of ULP maturation, Ub removal, and editing of poly-Ub chains, aid in this Ub removal process [126]. Most DUBs exhibit substrate-specificity, although some break down Ub chains in a non-specific way. The five conserved families of DUBs in humans are JAMM, UCH, USP, OTU, and MJD. With the exception of the JAMM zinc metalloproteases, all DUBs are cysteine proteases [112]. Rpn11, a 19S RP lid component, has de-ubiquitinating properties. Protease-interacting proteins (PIPs) are additional DUBs that interact with the complex but are not essential components of the proteasome [127]. Among these, both yeast and humans have Ubp6/USP14 and Uch2/Uch37. There is an interaction between Uch2/Uch37 and the proteasomal Ub receptor Rpn13. It is possible that Ubp6/USP14 and Uch2/Uch37 serve as “proof-reading” enzymes, whose job it is to eliminate Ub chains from proteins that the UPS mistakenly encounters and ubiquitinates [128]. Two other investigations found 18 and 29 DUBs in *P. falciparum*, respectively [110,129]. *P. falciparum* has been found to harbor homologs of human UCH37 (*Pf*UCH54) and UCHL3 (*Pf*UCHL3). They are known to exhibit deNeddylating and deubiquitinating properties [130,131,132]. A putative homolog of human USP14, known as PFE1355c, is also found in *P. falciparum*, exhibiting 33% sequence identity (Table 4). Given their inherent protease activity, plasmodial DUBs could serve as promising targets for antimalarial drug development [69]. PR169, a more recently developed DUB inhibitor, exhibits high selectivity for the β-5 subunit and has been observed to induce apoptosis in various cancer cell lines. Moreover, its potential application in malaria treatment is under investigation, given its potent proteasome inhibition capabilities. WP1130, another novel DUB inhibitor, has shown significant anti-parasitic activity and demonstrated efficacy against *P. falciparum* and *P. berghei* in preclinical studies, inhibiting intraerythrocytic development and reducing parasite load [18]. Because of their function in hemoglobin breakdown, parasite cysteine proteases have attracted attention [133]. The effects of anti-plasmodial cysteine protease inhibitors and currently available DUB inhibitors (such Ubal, UbVS, or cyclopentenon) on Ub-protein accumulation may be better understood by carrying out extensive research in malarial parasites [134]. Since there is no evidence of deNeddylating activity for the mammalian homologs of *Pf*UCH54 and *Pf*UCHL3, these two DUBs’ deNeddylating capabilities may be particularly interesting for drug design.

We used *Plasmodium falciparum* 3D7 genomes from PlasmoDB, an online server (https://plasmodb.org/plasmo/app) (accessed on 21 August 2024) as queries in a BLAST search to find parasite homologs of proteasomal subunits by comparing them to the human genome (from UniProt) [135]. Based on high-scoring scores, predicted homologs of the proteasome subunit sequences were sorted. The majority of the human 26S proteasome subunits have plasmodial homologs, according to this computational investigation (Table 3). Although more biochemical research is required to establish the similarity of contacts and proteolytic activity of the subunits, these in silico findings offer preliminary support for the similarity of the 26S proteasome in humans and malarial parasites.

### 3.5. Advancements in Proteasome Inhibitor Design for Malaria

The recent structural information for the *P. falciparum* proteasome has spurred renewed efforts to design novel inhibitors and re-evaluate the seven classes of proteasome inhibitors: lactacystins, salinosporamides, epoxyketone, sulfonyl fluorides, boronic acids, cyclic peptides, and AsnEDA and derivatives. Compounds from seven different classes will be discussed after being tested against *Plasmodium* individually. This will be followed by a summary of the use of related compounds in the treatment of various parasitic infections.

#### 3.5.1. Lactacystins and Salinosporamides

Lactacystin was the first natural proteasome inhibitor to be identified and tested for anti-plasmodial action [136,137]. It inhibits chymotrypsin-like, trypsin-like, and peptidyl glutamyl peptide-hydrolyzing activities by binding irreversibly to all catalytic β subunits [90,137]. Using a mouse malaria model, lactacystin has shown efficacy against the pre-erythrocytic and erythrocytic stages of malaria both in vitro and in vivo [95,136]. The in vitro treatment of sporozoites with lactacystin prevents the main rRNA pool in HepG2 cells from changing from C-type (sporozoites) to A-type (EEF stage), which is required for effective mammalian host proliferation. Additionally, it prevents trophozoite-to-schizont transformation, which leads to problems in the commencement of DNA synthesis and inhibits erythrocytic phases. Treating schizonts causes defects that stop them from rupturing, which might suggest issues with cell cycle regulation or the blocking of proteases needed for their release. However, lactacystin’s clinical therapeutic potential is limited due to its uneven inhibitory effects and lack of selectivity, which results in toxicity at the levels needed for parasite clearance.

**Figure 4:**
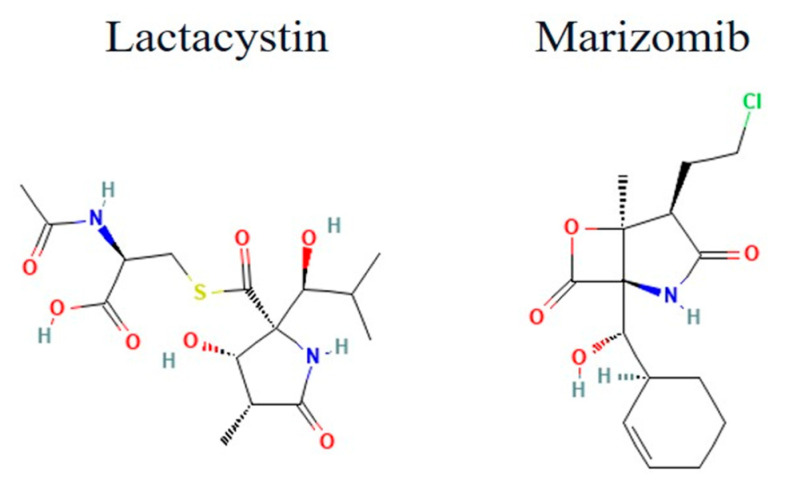
The 2D structure of lactacystin and salinosporamide (Marizomib).

The marine actinomycete Salinispora tropica was found to have a novel β-lactone called salinosporamide A (Marizomib). With an IC_50_ of 11.4 nM against *P. falciparum*’s erythrocytic stages, it demonstrates noticeably greater potency [92,138]. According to Prudhomme et al., this substance prevents the advancement of all erythrocytic stages, including schizont rupture, and following treatment, parasite extracts show an increase in ubiquitinated proteins. A dosage of 130 μg/kg of salinosporamide A administered in vivo to infected mice dramatically decreased P. yoelii parasitemia. Its toxicity at effective dosages is a significant worry despite its efficacy; therefore, more optimization is required to create synthetic compounds with higher parasite selectivity (Figure 4) [92].

#### 3.5.2. Epoxyketone Derivatives

Targeting the chymotrypsin activity of the β-5 subunit, these peptide derivatives are usually very strong and highly selective inhibitors that bind the proteasome permanently. Eponemycin and epoxomicin, the first naturally occurring epoxyketones, were identified in the early 1990s through screening directed at targeting melanoma cells [139,140]. This class of peptides originating from actinomycetes has expanded since its discovery to encompass a wide range of peptides with varying lengths and modifications. At low doses, epoxomicin demonstrated the ability to effectively kill erythrocytic stages [86,95,96,141] and gametocytocidal activity as low as 0.1 μM [86]. A new study demonstrated that epoxomicin’s anti-plasmodial activity was independent of the *Kelch13* genotype in a number of field isolates, indicating its potential use in combination with ACTs [142,143,144]. However, A549 and NIH 3T3 cell viability is reduced by 20% after treatment with 1 μM epoxomicin, suggesting toxicity problems. This emphasizes how important it is to create analogs that specifically target the *Plasmodium* proteasome.

**Figure 5:**
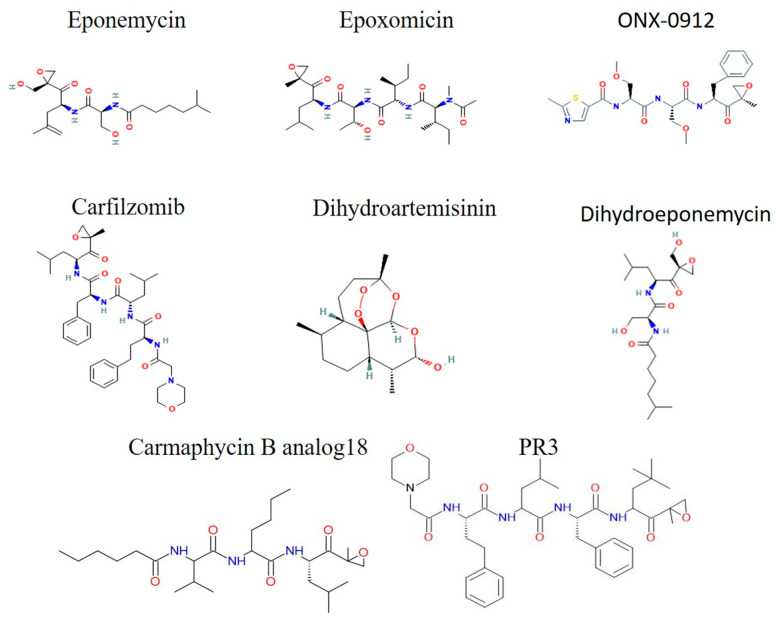
The 2D structures of the Epoxyketone Derivatives drugs.

Dihydroeponemycin, a synthetic derivative of eponemycin, was found to be potent against E2 and E3 in silico with a binding affinity of −3.8 and −4.1, respectively [85]. Moreover, one of the synthetic analogs of epoxomicin, YU101, inhibits erythrocytic stages at nanomolar level concentrations and Proteolix (now Onyx) exploited it as a lead compound in a screening that led to the development of carfilzomib (PR171, Kyprolis). In 2012, carfilzomib was authorized by the FDA as the second proteasome inhibitor to treat multiple myeloma. Carfilzomib, an antimalarial medication candidate, demonstrated minimal effectiveness against asexual stages but no toxicity at 1.5 mg/kg in the *P. berghei* mouse model [84,145]. Crucially, carfilzomib and dihydroartemisinin (DHA) complemented each other even at lower dosages. A selective inhibitor that kills both asexual stages and gametocytes at nanomolar concentrations without harming human foreskin fibroblast cells at its solubility limit (50 μM) was found through the additional high-throughput screening (HTS) of 670 carfilzomib analogs for the antimalarial drug compound PR3. This is because it could not bind and inhibit the human proteasome β-2 subunit. Non-toxic PR3 treatment also somewhat postponed the development of *P. berghei* parasitemia in infected mice [84,146]. Carmaphycin B, a natural tripeptide having an α’,β’-epoxyketone group at its C-terminus, was screened for 20 synthetic analogs. Analog-18 was found to have strong antimalarial activity against both gametocytes and asexual stages. Compared to carmaphycin B, analog-18 has a therapeutic index 100-times higher than carmaphycin B, effective at lower concentrations with a greater safety margin. It also has an IC_50_ of approximately 3 nM against the asexual stages of the parasite [79,147]. This finding illustrates that minor structural variations between the proteasomes of the parasite and humans can be leveraged to enhance the selectivity and reduce the toxicity of proteasome inhibitors (Figure 5).

#### 3.5.3. Sulfonyl Fluorides

First produced and assessed in 1995, sulfonyl fluorides are potent, irreversible inhibitors of calpain and cysteine proteases. Their proteasome blocking action was documented in 1997 [148,149]. By attaching themselves to and deactivating the β-1 and β-5 subunits of the *Plasmodium* proteasome, sulfonyl fluorides target this enzyme [150]. They also demonstrated how PW28, a lead sulfonyl fluoride chemical, efficiently stops gametocyte development at all stages without compromising commitment and inhibits the development of asexual stages regardless of when therapy is initiated. When tested at 500 μM, the majority of the compounds showed no in vitro toxicity. However, despite a notable reduction in parasitemia, *P. berghei*-infected mice treated with 10 mg/kg of PW28 showed indications of harm. Based on parasite-specific peptide cleavage studies, the latest synthesis of WLL-vs highlights the potential to expand the therapeutic window of sulfonyl fluoride drugs [102].

#### 3.5.4. Peptide Aldehydes and Boronic Acids (Non-Peptide Small Molecules)

**Figure 6:**
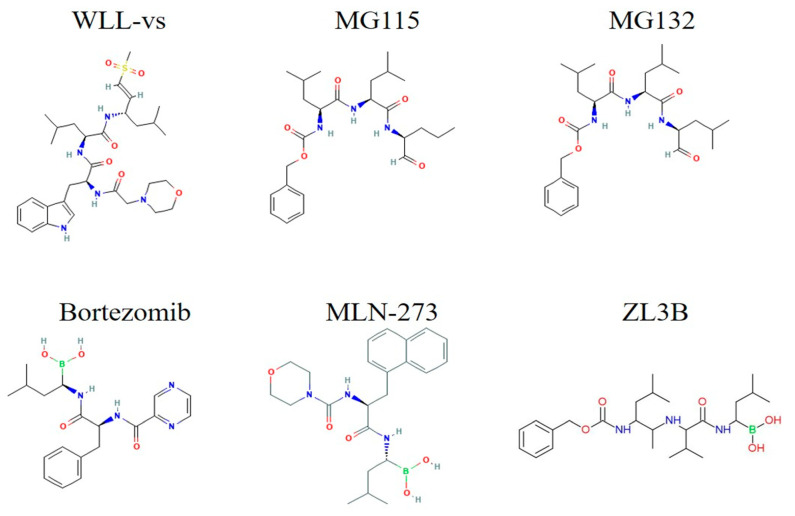
The 2D structures of the Sulfonyl Fluoride drugs.

The early 1990s saw the first reports of peptide aldehydes inhibiting proteasome activity [151,152]. Subsequent studies demonstrated that the peptide aldehyde derivatives MG115 and MG132 have a potent inhibitory impact on proteasomes because they reversibly bind to and block the chymotrypsin activity of the β-5 subunit of the human proteasome [96,153,154]. A derivative of boronic acid, bortezomib binds to the human proteasome’s β-5 subunit reversibly and inhibits it [83,155,156,157]. In 2003, it was the first proteasome inhibitor authorized by the FDA to treat multiple myeloma [158,159,160,161]. The first derivative of dipeptidyl boronic acid to be tested for action against *Plasmodium* parasites was MLN-273, a bortezomib analog with a longer half-life [93,162]. Studies demonstrate that MLN-273 inhibits erythrocytic development of *P. falciparum* in the ring stage and reduces *P. berghei*’s exo-erythrocytic development in vitro into schizonts (100 nM concentration). At 100 nM, MLN-273 has little effect on HepG2 cells; but, at 1000 nM, it causes HepG2 cells to undergo apoptosis [93,163]. Bortezomib and its analog ZL3B were investigated for their anti-parasitic properties in a prior study, which showed that they inhibited intraerythrocytic development before DNA synthesis without influencing parasite egress [83]. ZL3B and bortezomib were found to have IC_50_ values between 30 and 40 nM. Despite conflicting opinions regarding the sensitivity of drug-sensitive and drug-resistant parasites to bortezomib, co-treatment of *P. falciparum* revealed a potent synergistic impact between DHA and bortezomib [83,95,142]. Notwithstanding these developments, more research is necessary to determine the toxicity of boronic acid derivatives. In the hunt for strong and specific antimalarial drugs, the advent of novel boronic acid derivatives, some of which are undergoing clinical trials, makes this group of proteasome inhibitors a viable option (Figure 6) [141,164].

#### 3.5.5. Cyclic Peptides

**Figure 7:**
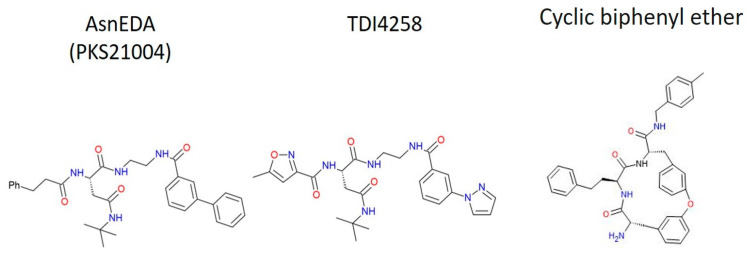
The 2D structure of AsnEDA, TDI4258, and cyclic biphenyl ether.

A comparatively recent type of naturally occurring proteasome inhibitor, cyclic peptides are obtained from the fungus *Apiospora montagnei*. The only known proteasome inhibitor that non-covalently binds and inhibits all three β subunits is the cyclic peptide TMC-95A. Due to their greater availability and lack of persistent sequestration in the red blood cell proteasome, non-covalent inhibitors are beneficial in the treatment of malaria. Cyclic peptide-1, a TMC-95A derivative, was discovered by researchers after screening a library of 1600 non-covalent proteasome inhibitors. In vitro, the cyclic biphenyl ether compound exhibited greater specificity for the Plasmodium proteasome compared to the human proteasome with an IC_50_ of 35 nM (Figure 7) [165,166]. As with WLL-vs, the co-inhibition of the β-2 and β-5 subunits is responsible for cyclic peptides’ selectivity [103]. According to additional research, cyclic peptide pulse therapy for one hour prevented *P. falciparum’s* erythrocytic cycle from progressing at any stage of the parasite’s growth. A subsequent investigation revealed that the proteasome’s selectivity over cysteine proteases in the cell is much enhanced when peptide aldehydes are given a macrocyclic structure. This discovery raises the possibility that cyclic peptide compounds could be further optimized to improve their efficacy and selectivity against the *P. falciparum* proteasome [167].

#### 3.5.6. AsnEDA and Derivative

Asparagine ethylene diamines (AsnEDAs) are peptidomimetic proteasome inhibitors that specifically and reversibly suppress the β5 activity of proteasome. Their hydrophilic properties enhance selectivity for the parasite proteasome, boosting their anti-parasitic effectiveness. In one study, WHZ-13, derived from the parent molecule PKS21004, demonstrated the highest potency, with an IC_50_ of 4.7 nM and strong selectivity over the constitutive β5c and immunoproteasome β5i subunits [168]. Further optimization led to TDI4258, a standout inhibitor with over 1000-fold selectivity for parasite proteasome compared to mammalian HepG2 cells [169]. TDI4258 also exhibited synergy with DHA and showed activity against various artemisinin-resistant parasite strains and field isolates from Uganda. However, a challenge in studying these inhibitors is that *Plasmodium* β2 subunits can partially process β5 substrates, which specifically target β5 activity reversibly. Additionally, this class of inhibitors has a short half-life of 30 min and shows limited effectiveness when used alone in the *P. yoelii* malaria model. However, when administered at 15 mg/kg in combination with 60 mg/kg of WLW-vs, the compound demonstrated efficacy (Figure 7) [169].

## 4. Future Directions

The plasmodial proteasome has emerged as a promising candidate for multi-stage drug development against malaria. This advancement is rooted in the progress made in the field of proteasome inhibition for cancer treatment. For instance, bortezomib, a proteasome inhibitor developed for multiple myeloma, has shown efficacy in targeting rapidly dividing cells, suggesting its potential utility in managing the blood stages of *Plasmodium*. However, the challenge remains in achieving a high therapeutic index due to the need for specificity towards *Plasmodium* and the effective targeting of less accessible stages such as the liver and gametocytes.

Our capacity to find and create selective proteasome inhibitors has been greatly improved by recent technical developments. In this procedure, HTS has become an essential tool that enables the quick assessment of sizable chemical libraries to find possible inhibitors. For instance, a recent study combined structure-based drug design with HTS to discover new classes of proteasome inhibitors. These inhibitors specifically target the plasmodial proteasome by taking advantage of variations in substrate binding and protease activity between the human and plasmodial proteasomes [12,31].

Structure-based medication design has also been very advantageous for the development of selective inhibitors [170]. Researchers have discovered distinct binding locations and structural characteristics that can be used to create highly selective inhibitors by interpreting the crystal structures of the plasmodial proteasome. One study, for instance, used molecular docking and X-ray crystallography to design a set of proteasome inhibitors that showed selectivity for the plasmodial proteasome over its human counterpart [103,171]. This technique increases specificity while simultaneously improving specificity.

Proteomics based on mass spectrometry has also provided important information about the substrate profile of the proteasome in *Plasmodium* [172]. The exact mapping of ubiquitinated substrates is important to comprehend the various biological processes that the plasmodial proteasome controls, and recent advancements in this technology have made this possible. This information has led to the development of inhibitors that can selectively disrupt a variety of processes. For example, a study that employed quantitative mass spectrometry to determine proteasome substrates and clarify the UPS’s function in Plasmodium life-cycle stages resulted in the discovery of novel, highly selective inhibitors [173].

Protease inhibitor development can be further enhanced by computational techniques such as molecular dynamics simulations [171]. Through the dynamic modeling of inhibitor–plasmodial proteasome interactions, these simulations provide researchers with insights into binding kinetics and potential resistance mechanisms [174].

Recent research has shown the potential of species-selective proteasome inhibitors. A study on *Mycobacterium* TB, for instance, found a proteasome inhibitor that was highly selective for the bacterial proteasome, providing a model for creating drugs that are similar to those that are used against *Plasmodium* [175].

To summarize, the combination of HTS, computational modeling, mass spectrometry-based proteomics, and structure-based drug design has improved the ability to target the UPS in *Plasmodium.* These technologies may help in the development of novel, more effective antimalarial drugs by advancing our understanding of the cell biology and biochemistry of the proteasome in multi-stage parasites.

## 5. Conclusions

This review underscores the critical role of the ubiquitin-proteasome system (UPS) as a multifaceted target in the development of novel antimalarial drugs. The global escalation of antimalarial drug resistance, particularly to combination therapies, highlights the necessity for innovative treatments with unique mechanisms of action. Resistance in *Plasmodium falciparum* and *Plasmodium vivax* is notably prevalent in regions such as South America, Africa, and Southeast Asia. The complex life cycle of the malarial parasite presents significant challenges in drug discovery. However, targeting various components of the UPS, including E1/E2/E3 ligases, deubiquitinases, and the 20S proteasome, offers promising avenues for intervention. Inhibitors such as MLN4924 and carmaphycin B have demonstrated potent antimalarial activity by disrupting UPS functions, leading to the accumulation of ubiquitinated proteins and induction of parasite death. Moreover, combining UPS inhibitors with existing antimalarial agents like dihydroartemisinin has shown synergistic effects, enhancing efficacy against both sensitive and resistant strains. These findings emphasize the UPS’s potential as a strategic target in combating malaria and overcoming current therapeutic challenges.

## Figures and Tables

**Table 1:** Comparative analysis of key UPS components for antimalarial drug development versus human disease targets.

Component	Target in Parasites (Antimalarial)	Target in Humans (Diseases)	Challenges	Ref.
Ubiquitin	Disrupt ubiquitin tagging to impair parasite protein homeostasis.	Target ubiquitin-activating enzymes in cancer therapy.	High conservation limits selectivity.	[63,64]
E3 Ligases	Inhibit parasite-specific E3 ligases to block protein degradation.	Target E3 ligases (e.g., MDM2) in cancer and inflammation.	Functional redundancy complicates specificity.	[65,66]
Proteasome	Use proteasome inhibitors (e.g., Epoxyketones) selective for *Plasmodium*.	FDA-approved inhibitors (e.g., Bortezomib) for cancer therapy.	Toxicity due to effects on normal human cells.	[67,68]
DUBs	Inhibit *Plasmodium*-specific DUBs to induce proteotoxic stress.	Target DUBs (e.g., USP7, USP14) for cancer and neurodegeneration.	High conservation; off-target effects must be minimized.	[69,70]

**Table 2:** The drugs targeting the malarial 20S proteasome.

Compounds	IC_50_ (µM, In Vitro)	Pharmacokinetics		Class	Water Solubility		Ref
		GI Abs	BBB Perm		Sol	Class	
Bortezomib	0.031–0.043 (*Pf*3D7)	High	No	Peptide boronate	7.53 × 10^−1^	Soluble	[83]
Carfilzomib	0.025 (*Pf*)	Low	No	Epoxyketone	1.03 × 10^−3^	Moderately soluble	[79,84]
Dihydroeponemycin	In silico study	High	No	Epoxyketone	3.53 × 10^0^	Soluble	[85]
Epoxomicin	0.054 (gametocyte) 0.041 (asexual stages)	Low	No	Epoxyketone	2.04 × 10^−1^	Soluble	[86,87]
Gliotoxin	2.17 (*Pf*)	High	No	Non-covalent, reversible	1.48 × 10^1^	Very soluble	[88,89]
Lactacystin	1.2–1.5 (*Pf* blood stages)	Low	No	Β-lactone	1.47 × 10^1^	Very soluble	[90,91]
Marizomib, NPI-0052, Salinosporamide A	0.0114 (*Pf*)	High	No	Β-lactone-γ-lactam	6.84 × 10^−1^	Soluble	[92,93,94]
MG-115	0.0975 *(Pf*3D7)	High	No	Peptide aldehyde	1.78 × 10^−2^	Moderately soluble	[95]
MG-132	0.0476 (*Pf*)	High	No	Peptide aldehyde	8.00 × 10^−3^	Moderately soluble	[96]
ONX-0914 (PR-957)	n.d	High	No	β-lactone	1.07 × 10^−1^	Soluble	[97,98]
TDI-8304	3.1 (hypnozoite stage)	High	No	Vinyl sulfone	2.17 × 10^−2^	Moderately soluble	[22,99]
Tripterin (celastrol)	0.50–0.82µM (asexual blood stage *Pf*)	Low	No	Triterpenoid	2.21 × 10^−4^	Poorly soluble	[100,101]
WLL_vs	0.011–0.013 (against diverse strains)	Low	No	Vinyl sulfone	2.18 × 10^−2^	Moderately soluble	[102,103]
YU-101	0.0245 (*Pf*3D7)	n.d	n.d	Epoxyketone	n.d	n.d	[95]
ZL_3_B	0.04 (*Pf*3D7)	High	No	Peptide boronate	5.50 × 10^−3^	Moderately soluble	[83]

GI Abs: gastrointestinal absorption; BBB perm: blood–brain permeability; Sol: solubility mg mL; n.d: no data; *Pf*, *Plasmodium falciparum*; water solubility level: very soluble (Log S > 0), soluble (>Log S 0 to −2), moderately soluble (>Log S −2 to −4), poorly soluble (>Log S −4 to −6).

**Table 3:** Drugs targeting the malarial E3 ligase.

Compounds	IC_50_ (µM, In Vitro)	Pharmacokinetics		Note	Water Solubility		Ref.
		GI Abs	BBB Perm		Sol	Class	
HLI 373	2.36 (CQ-S), 3.47 (CQ-R)	High	Yes	HDM2 inhibitor	1.75 × 10^−2^	Moderately soluble	[111]
JNJ-26854165	2.17 (CQ-S), 1.86 (CQ-R)	High	Yes	Modulate ubiquitin proteasome pathway	1.06 × 10^−2^	Moderately soluble	[111]
MI-219	n.d	High	No	MDM2 inhibitor	3.35 × 10^−3^	Moderately soluble	[122]
MI-63	0.58	High	No	MDM2 inhibitor	8.94 × 10^−4^	Moderately soluble	[91]
Nutlin-3A	12.76 (CQ-S), 18.56 (CQ-R)	High	No	MDM2 inhibitor	1.73 × 10^−4^	Poorly soluble	[111]
Oridonin	2	High	No	Activate E3 ubiquitin ligase	2.58 × 10^0^	Soluble	[123]
SMER 3	12.06 (CQ-S), 20.58 (CQ-R)	High	Yes	Inhibitor of a yeast SCF family E3 ubiquitin ligase	5.08 × 10^−1^	Soluble	[111]
Thalidomide	n.d	High	No	Immunomodulatory	3.94 × 10^0^	Very soluble	[111,124,125]

GI Abs: gastrointestinal absorption; BBB perm: blood–brain permeability; Sol mg/mL: solubility mg mL; n.d: no data; CQ-S: chloroquine-sensitive strain; CQ-R: chloroquine-resistant strain; water solubility level: very soluble (Log S > 0), soluble (>Log S 0 to −2), moderately soluble (>Log S −2 to −4), poorly soluble (>Log S −4 to −6).

**Table 4:** The amino acid sequence lengths of both human and malarial proteasome subunits are represented with their sequence similarity.

Proteasome Subunits	*H. sapiens* (GenBank)		*P. falciparum* (PlasmoDB)		Seq Identity (%)	E-Value
	Accession No.	Seq Length (AA)	Accession No.	Seq Length (AA)		
CP subunits						
α type 1	P25786	263	PF14_0716	254	44.0	7 × 10^−71^
α type 2	P25787	234	PFF0420c	235	57.7	6 × 10^−95^
α type 3	P25788	255	PFC0745c	252	36.2	1 × 10^−56^
α type 4	P25789	261	PF13_0282	246	53.4	8 × 10^−88^
α type 5	P28066	241	PF07_0112	256	54.4	4 × 10^−90^
α type 6	P60900	246	MAL8P1.128	260	44.2	4 × 10^−76^
α type 7	O14818	248	MAL13P1.270	241	51.1	1 × 10^−81^
β type 1	P20618	241	PFE0915c	240	43.2	1 × 10^−59^
β type 2	P49721	201	PF14_0676	195	40.2	9 × 10^−46^
β type 3	P49720	205	PFA0400c	218	44.0	4 × 10^−65^
β type 4	P28070	264	MAL8P1.142	265	36.3	7 × 10^−49^
β type 5	P28074	263	PF10_0111	271	53.5	7 × 10^−78^
β type 6	P28072	239	PFI1545c	282	28.5	6 × 10^−36^
β type 7	Q99436	277	PF13_0156	270	55.8	7 × 10^−95^
**RP subunits (base)**						
RS 4 or RPT2	P62191	440	PF10_0081	448	76.6	
RS 6A or RPT5	P17980	439	PF11_0314	439	71.3	
RS 6B or RPT3	P43686	418	PFD0665c	392	68.6	
RS 7 or RPT1	P35998	433	PF13_0063	420	74.7	
RS 8 or RPT6	P62195	406	PFL2345c	435	77.3	
RS 10B or RPT4	P62333	389	PF13_0033	393	68.9	
Non-ATPase RS 1 or RPN2	Q99460	953	PF14_0632	1172	38.5	
Non-ATPase RS 2 or RPN1	Q13200	908	PFB0260w	966	36.1	
Non-ATPase RS 4 or RPN10	P55036	377	PF08_0109	481	40.9	7 × 10^−35^
Non-ATPase RS RPN13	Q16186	407	PF14_0138	253	33.8	1 × 10^−17^
**RP subunits (lid)**						
Non-ATPase RS 3 or RPN3	O43242	534	MAL13P1.190	503	38.8	1 × 10^−100^
Non-ATPase RS 4 or RPN6	O00231	422	PF14_0025	666	35.9	2 × 10^−28^
Non-ATPase RS 6 or RPN 7	Q15008	389	PF11_0303	393	38.3	1 × 10^−102^
Non-ATPase RS 7 or RPN8	P51665	324	PFI0630w	338	44.7	3 × 10^−74^
Non-ATPase RS 8 or RPN 12	P48556	350	PFC0520w	304	35.0	1 × 10^−33^
Non-ATPase RS 12 or RPN5	O00232	456	PF10_0174	467	38.6	8 × 10^−101^
Non-ATPase RS 13 or RPN9	Q9UNM6	376	PF10_0298	393	26.3	8 × 10^−37^
Non-ATPase RS 14 or RPN 11	O00487	310	MAL13P1.343	311	63.0	2 × 10^−138^
Non-ATPase RS RPN 15 or DSS1	P60896	70	MAL7P1.117	106	61.0	

CP, core proteasome; RP, regulatory proteasome; RS, regulatory subunit; E-value, expectation value; AA, amino acids.

## Data Availability

All of the data are available in the manuscript.
